# Reducing stroke burden through a targeted self-management intervention for reducing stroke risk factors in high-risk Ugandans: A protocol for a randomized controlled trial

**DOI:** 10.1371/journal.pone.0251662

**Published:** 2021-06-22

**Authors:** Mark Kaddumukasa, Josephine Najjuma, Scovia Nalugo Mbalinda, Martin N. Kaddumukasa, Jane Nakibuuka, Christopher Burant, Shirley Moore, Carol Blixen, Elly T. Katabira, Martha Sajatovic

**Affiliations:** 1 Department of Medicine, School of Medicine, College of Health Sciences, Makerere University, Kampala, Uganda; 2 Department of Nursing, Mbarara University of Science and Technology, Mbarara, Uganda; 3 Department of Nursing, College of Health Sciences, Makerere University, Kampala, Uganda; 4 Department of Medicine, Mulago Hospital, Kampala, Uganda; 5 Louis Stokes VA Medical Center, Geriatric Research Education, and Clinical Center, Cleveland, OH, United States of America; 6 Frances Payne Bolton School of Nursing, Case Western Reserve University, Cleveland, OH, United States of America; 7 Neurological and Behavioral Outcomes Center, University Hospitals Cleveland Medical Center & Case Western Reserve University School of Medicine, Cleveland, OH, United States of America; PLoS ONE, UNITED STATES

## Abstract

**Introduction:**

Stroke burden is rapidly increasing globally. Modifiable risk factors offer an opportunity to intervene, and targeting hypertension is a key actionable target for stroke risk reduction in sub-Saharan Africa. This 3-site planned randomized controlled trial builds on promising preliminary data.

**Methods:**

A total of 246 Ugandan adults will be recruited randomized to experimental intervention vs. enhanced treatment control. Intervention participants will receive six weekly group-format stroke risk reduction self-management training sessions, and the controls will receive information on cardiovascular risk. The primary study outcome is systolic B.P. measured at baseline, 13-week, 24 weeks (6 months). Secondary outcomes include other biological and behavioral stroke risk factors.

**Discussion:**

The curriculum-guided self-management TargetEd MAnageMent Intervention (TEAM) program is anticipated to reduce the stroke burden in Uganda.

**Trial registration:**

ClinicalTrials.gov identifier: NCT04685408, registered on 28 December 2020.

## Introduction

Chronic non-communicable diseases (NCDs) are increasing globally, with a disproportionate burden on low-middle income countries (LMICs). Approximately 80% of NCD deaths occur in developing countries, with nearly a third of the deaths occurring in those aged less than 60 years compared to 13% in high-income countries [1]. Stroke contributes substantially to NCD-related disability and is attributed to 16.8% of deaths in LMICs, compared with 13.2% of deaths in high-income countries [1].

In Uganda, stroke burden is growing and affecting relatively young individuals where it is a severely neglected condition [2, 3], despite accounting for 15% of hospital admissions and is a major contributor to mortality [4–9]. Risk factors for stroke, which were once rare in traditional African societies, are becoming a major public health problem [10]. The adaptation of western cultural behaviors such as sedentary lifestyle, tobacco and alcohol use, high fatty, and salty diets all contribute to stroke risk [11]. A rapidly increasing aging population and urban migration are also associated with stroke risk. The overall prevalence of hypertension in Uganda is relatively high at 26.4% of adult Ugandans [12]. However, many risk factors are potentially modifiable, which presents an opportunity to reduce the stroke burden. Hypertension is a key actionable target for stroke risk reduction in Sub-Saharan Africa (SSA), as are other potentially modifiable lifestyle factors such as obesity and smoking.

An approach that engages people at risk for stroke and includes key social and cultural elements may be particularly salient for future generalizability and scale-up. Building upon promising pilot work, this 3-site, prospective randomized controlled trial (RCT) will test a curriculum-guided self-management TargetEd MAnageMent Intervention (TEAM) program [13] designed to help reduce stroke burden in Ugandan adults vs. an Enhanced Treatment As Usual (ETAU) control comparison. We hypothesize that individuals in TEAM will have significantly reduced systolic B.P., serum cholesterol, and improved glycemic control among diabetic participants compared to individuals randomized to ETAU.

## Materials and methods

### Overview

This methods overview describes a 2-phase study that will use input from relevant stakeholders to refine the TEAM intervention to ensure acceptability (Phase 1) and then test the intervention’s effects in reducing stroke risk among a high-risk stroke population (Phase 2). The project will be operationalized in three specific objectives:

### Study objectives

1. Refine the TEAM curriculum for optimal acceptability and integration in the Ugandan setting guided by stakeholders’ input (patients/family, clinicians, administrators).
2. Conduct a randomized controlled trial comparing the efficacy of TEAM vs. enhanced treatment as usual (ETAU) in 246 Ugandans (TEAM, N = 123; ETAU, N = 123) at high risk for stroke.
3. Identify barriers and facilitators to TEAM implementation to inform subsequent scale-up and spread qualitative methods and guided by a conceptual implementation model.

### Study setting

This study’s 3-enrolling sites are Kiruddu and Nsambya hospitals in Kampala and Mbarara Hospital in Western Uganda. Kiruddu hospital has a neurology unit with a 40-bed inpatient section and receives approximately 60 stroke patients a month. Nsambya Hospital, with a capacity of 361 beds, receives about 15 new stroke patients every month and will offer a suburban population for the study. Mbarara hospital in Western Uganda provides neurology care for about 10 patients per month. All hospitals provide outpatient neurology clinics where stroke survivors attend for their long-term rehabilitation and care.

### Study design

#### Phase 1

**Fig 1:**
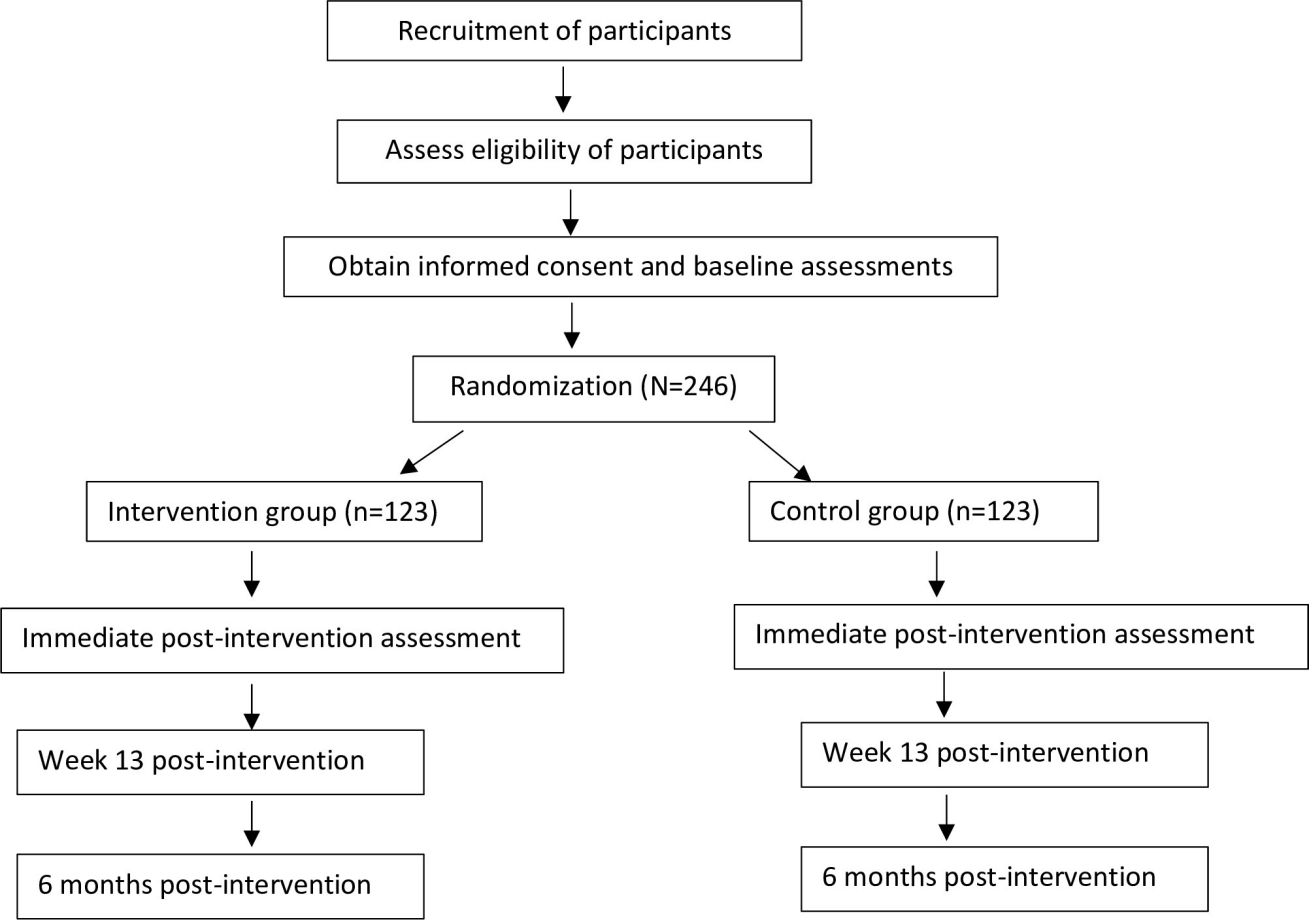
Study flow diagram.

We will refine the TEAM intervention for content and process guided by stakeholders (patients/family, clinicians, administrators) into the local context in year 1. A stakeholder advisory board (SAB) will guide modest refinement of the program to meet diverse stakeholder needs and integrate TEAM with clinic workflows. Phase 2: This is a prospective 6-month randomized controlled trial (RCT) that will evaluate the effects of standard medical care + TEAM vs. enhanced medical treatment as usual (ETAU) on key stroke risk factors. Fig 1, showing the flow diagram of the study progress through the phases of a parallel randomised trial of two groups (that is, enhanced treatment as usual and TEAM intervention).

The study was registered on 28^th^ December 2020 at ClinicalTrials.gov identifier: NCT04685408, https://clinicaltrials.gov/ct2/show/NCT04685408.

### Study population

#### Phase 1

The stakeholder advisory board (SAB) will be composed of up to 18 relevant stakeholders, including stroke survivors, individuals with multiple stroke risk factors, and family members of individuals at risk for stroke and stroke survivors, clinicians, and administrators who practice in the proposed study enrollment sites.

#### Phase 2

The study participants will be adults aged 18 years or more who are at risk for stroke as defined by the following inclusion criteria:

- High systolic blood pressure (BP) defined as >140 mmHg assessed on at least two occasions at least three days apart and either criterion b or c as noted below:
- At least one other modifiable stroke risk factor including diabetes, hyperlipidemia, obesity, smoking, problem alcohol use, or sedentary lifestyle.
- History of stroke or transient ischemic attack within the past five years. In addition to the inclusion criteria noted above, individuals must be able to participate in groups and study procedures and provide informed consent to study participation.

*Exclusion criteria*. Individuals with sickle-cell disease, pregnant or lactating women, and individuals with dementia will be excluded. Brief dementia screening will be conducted with the Identification for Dementia in Elderly Africans (IDEA) previously tested in SSA samples [14].

#### Phase 1 specific procedures

Patient, family, and clinician/administrator representation will be balanced across recruitment sites. There will be three meetings with video-conference call services available for those who cannot be physically present during the first six months of the project (using Zoom or a similar video conferencing platform). In the first call, SAB members will review the TEAM curriculum and identify content areas they may need to edit or add. The study team members will then make these modifications to the TEAM intervention manual. It is expected that at least some of the added content will be appropriate to compile a supplemental content "toolkit" that can be used as needed based on patient needs. In the 2^nd^ meeting, SAB members will review the revised TEAM content and make any additional /final suggestions. In the 3^rd^ meeting, the SAB will be asked to identify strategies that will help integrate TEAM into clinic workflow. SAB meetings will be audio-recorded and assessed qualitatively. After Phase 1 is concluded, the SAB will continue to meet annually for years 2–4 and twice annually in year 5.

#### Phase 2 specific procedures

Specific procedures in the RCT are outlined in Table 1.

**Table 1:** Specific procedures to be conducted in a Ugandan stroke risk reduction RCT.

Procedures	Screen	Baseline	Week 1–12	Week 13	Week 13 -Month 6	Month 6
**Indentification of participants:**	X					
Inclusion/Exclusion criteria review						
Informed consent	X	X	X	X	X	X
Demographics		X				
Medical status & burden		X				
Self-reported Charlson Comorbidity Index		X		X	X	X
Medications		X				
Personal and family stroke history		X				
Randomization		X				
Primary outcome: systolic BP		X		X		X
**Secondary Outcomes:**		X		X		X
Diastolic B.P.; Serum cholesterol; HbA1c; Serum HDL, LDL, triglycerides; BMI; Diet questionnaire; GPAQ; GATS; AUDIT; General self-efficacy; INTERSTROKE stress; Medication adherence; Medication Access; Health resource use						
**Qualitative Assessment**		X		X		X
Patient						
Clinicians/Administrators						
SAB**						
**Intervention attendance**			X	X		X

#### Treatment randomization

After obtaining written, informed consent, and following all screening and study baseline procedures, individuals will be randomized on a 1:1 basis to participate in either TEAM or ETAU. The study participants will be blinded and data analysts. Block randomization with block sizes ranging randomly between 4 and 8 consecutive patients will be employed to ensure that equal numbers of TEAM and ETAU patients occur within strata and are balanced concerning relevant comorbidity (diabetes and previous stroke). The randomization list will be computer-generated by personnel within the biostatistics core of the CWRU Neurological and Behavioral Outcomes Center who are not members of the study staff.

### Intervention arm (TEAM)

The TEAM intervention self-management approach uses nurses and peer educator dyads (PEDs) composed of patients and their care partners to co-deliver the intervention. The TEAM will begin with one 30–60 minutes one on one orientation session, in which the nurse and PED meet with the patient and his/her care partner. This will be followed by six weekly, hour-long group sessions with 6–8 patients and their care partners, see Table 2: TEAM content/topics. To reinforce learning after the TEAM group sessions are completed, three brief (approximately 10–20 minute) monthly telephone calls will be made by the study nurse and to the patient over three months. These calls will support ongoing self-management and facilitate linkage with other care providers. All TEAM participants continue to receive treatment with their regular medical care providers.

**Table 2:** TEAM content/topics.

Session 1	Orientation and introductions, Empahsize ground rules, Establish a therapeutic realtionship, Discuss facts and myths about stroke, Overview and intercative discussion of stroke risk factors
Session 2	Medications to manage complications and reduce future rsik, Nutrition for best physical and emotional health, Healthy cooking and use of salt in food preparation
Session 3	Problem-solving skills and IDEA approach (Identify the probelm, Define possible solutions, Evaluate the solutions, Act on the best solution). Effects of excercise, smoking and other substances in recovery
Session 4	Medication routines, coping with stress and depression, Making healthy changes
Session 5	The importance of having a regular health care provider, Talking with health care providers, working with traditional healers
Session 6	A personal care paln to take care of the body and mind, Acknowledgement of group progress, Self managament and recovery as a lifestyle

### Control arm—Enhanced treatment as the usual arm (ETAU)

The ETAU arm will consist of an orientation session (30–60 minutes) with a study nurse who will provide patient-education materials on stroke risk and cover common risk factors such as hypertension, obesity, high salt/high-fat diet and diabetes. This visit will take place in the outpatient clinic, where participants get their medical care. Participants will be offered the opportunity to bring a family member to this visit who may also ask questions and assist them with understanding written materials for those with limited literacy. To control for the same number of patient contacts as TEAM, the nurse in ETAU will then follow-up with participants with a series of 9 brief phone calls spaced out over 6 months (approximately every two weeks during months 1 and 2, then approximately monthly thereafter). Content will reinforce materials provided in the orientation visit, and the nurse will be available to answer questions that may arise. Different nursing personnel will deliver the TEAM and ETAU interventions to minimize the chance of contamination across study arms.

### Qualitative evaluation

#### RCT participant in-depth interviews

Interviews on perceived barriers to stroke recovery and prevention as well as information on what community and care providers could do to increase stroke awareness and access to risk reduction support, will be conducted at baseline at each of the three sites.

The estimated sample size will include 30 patients from TEAM and 30 patients from ETAU (total N = 60). For qualitative interviews, this sample size is well above the recommended number of 20–50 individuals [15, 16]. The qualitative interview guide that was used to characterize participant perceptions in the TEAM pilot will be adapted for this RCT. The interview guide will also be structured to align with the integrated Promoting Action on Research Implementation in Health Services (i-PARIHS) framework [17]. We will balance the selection of respondents by age, gender, and diabetes comorbidity. All interviews will be audio-recorded and transcribed verbatim.

Textual data from these interviews will enrich our understanding of the processes that impact the RCT quantitative outcomes [18, 19]^,^ and could facilitate the timing of future study data collection for the analysis of mediators such as: What are the barriers to being able to follow TEAM recommendations and practices? Does change in health behaviors occur gradually, or are there specific points at which TEAM should be presented/taught? For the TEAM intervention, we will ask them to identify TEAM content elements that individuals felt was most salient in helping them to change health behaviors and to evaluate the importance of process elements, such as the group format and also evaluate how well the ETAU intervention matched perceived need for information and support.

#### Clinician and other staff qualitative evaluation

Clinicians who refer their patients to the study will be queried regarding their perception of the effect of the intervention on patient support needs at the 6-month time-point at the three sites. Physicians, nurses, pharmacists, and administrative staff will participate in qualitative interviews (N = 15 total, 5 at each site) that mirror the questions asked of patients and additionally on how they perceive TEAM as fitting in or being compatible with existing clinic workflow and recommendations on how this might be optimized in future scale-up.

*Study measures*. Demographic variables and existing medical burden (assessed with the self-reported Charlson Index [20]) will be evaluated at baseline, prior to study randomization. Baseline medical status will also be evaluated with personal and family stroke history as well as currently prescribed medications.

The primary outcome of the RCT will be a change in systolic B.P. from baseline to 6-month follow-up. Additional outcomes of interest will include diastolic B.P., cholesterol/lipids and glycosylated hemoglobin (HbA1c), body mass index (BMI), measures that evaluate diet, activity levels, substance use, self-efficacy, stroke knowledge, stress, medication adherence, medication access, and health resource use. Quantitative measures will be repeated at 13-week and 24-week (6-month) follow-up except for laboratory testing, which will be conducted only at baseline and 24-week follow-up. The qualitative evaluation will be conducted at baseline and 13-week follow-up.

#### Smoking status

Using the Global Adult Tobacco Survey (GATS) questionnaire, we will collect information on current tobacco smoking status, past daily smoking status for current less than daily smokers, and past smoking status. Other questions such as smokeless tobacco and secondhand smoke are also covered in the questionnaire [21].

#### Alcohol consumption

The Alcohol Use Disorders Identification Test (AUDIT) will evaluate substance use [22]. This includes questions on frequency, type of alcohol, and quantity consumed. Participants will be classified as engaging in potential problem alcohol use if they exceed the recommended level for safe alcohol intake, i.e., more than 3 drinks on average every time they drink, or if they undertook binge drinking (i.e., more than 3 drinks on one occasion in the one month preceding the evaluation. Total scores of 8 or more are recommended as indicators of hazardous and harmful alcohol use and possible alcohol dependence [22].

#### Fruit and vegetable intake

Lifestyle changes of interest will include diet and salt intake as measured by the modified dietary questionnaire [23]. It assesses fruit and vegetable consumption. To determine salt intake, participants will be asked, ’How often do you add salt to your cooking?’ and ’How often do you use salt at the table?’ (Never/rarely, Sometimes, Usually, Always).

#### Physical activity

Physical activity will be measured using the Global Physical Activity Questionnaire (GPAQ) [24]. It collects information on physical activity participation in three settings (or domains) and sedentary behavior. These domains are: activity at work, travel to and from places, and recreational activities. It has acceptable reliability and validity.

#### Psychological stress

We will assess psychological stress using a combined measure of general stress at home and in the workplace, adapting the stress instrument used in the INTERSTROKE study [25].

#### Self-efficacy and medication adherence

To assess risk factor management self-efficacy, we will use the General Self-Efficacy measure [23, 26]. Recognizing that adherence can be intentional or non-intentional, we will collect data on intentional non-adherence via the use of a standardized adherence attitudinal measure (Medication Adherence Report Scale /MARS) [27, 28] and query individuals on difficulty (financial or otherwise) in accessing medications or medical care.

#### Study procedures

All B.P. measurements will adhere to a standardized procedure [29]. Before the B.P. measurement study, participants will be requested beforehand to refrain from smoking and drinking alcohol or caffeinated beverages at least half an hour before the examination. B.P. will be measured with an automated sphygmomanometer. The participant will be asked to sit on a chair and rest quietly for 15 minutes with his/her legs uncrossed. The left-arm will then be placed on a table with the palm facing upward and the antecubital fossa at the level of the lower sternum. Two arm cuffs that fitted arm circumferences 9–13 inches and 13–17 inches will be available for use in B.P. measurement. Three measurements will be taken at least 5 minutes apart. The average of the last two readings will be considered as the final blood pressure reading.

#### Study outcomes

The primary study outcome is systolic BP measured at baseline, 13-weeks, and 24 weeks (6 months). Additional secondary outcomes include other biomarker variables ((HDL, LDL, triglycerides), diet, exercise, use of alcohol and tobacco, stress, and treatment adherence with risk-reducing medications), medication adherence, and health resource use. We will also explore associations of age, gender, urban vs. rural residential status, and stroke history (prior vs. no previous stroke) on TEAM outcomes.

#### Feasibility and fidelity

Attendance for each TEAM and ETAU contact will be recorded. Acceptability for each intervention will be assessed at 13 weeks with a brief self-rated survey. Following Fraser et al. [30], fidelity to the TEAM intervention will be assessed quantitatively and qualitatively. Fidelity to TEAM processes, content, and format, will be evaluated by random attendance of 20% of sessions by non-interventionist study staff to determine if sessions covered relevant TEAM constructs and health practices as identified in the specific sessions as well as the assessment of if written prompts were appropriately utilized, and if sufficient time was devoted to the question/answer/ comment session. Each fidelity dimension will be rated on a 1–10 scale.

### Quantitative data analysis

Preliminary descriptive analyses will examine change over time in systolic B.P., serum cholesterol, and serum HbA1c as well as secondary (biologic and behavioral) outcomes, including include other biomarker variables, stroke knowledge and attitudes, medication adherence, and health resource use. Primary and secondary outcome measurements will be assessed at baseline before randomization to either TEAM or ETAU and at the 13 week and 6-month time-points. Of primary interest is the treatment by time interaction in systolic B.P., measured from baseline to 6 months after randomization between TEAM and ETAU. Explanatory variables will include age, gender, stroke history, and rural vs. urban status.

The 13-week systolic B.P. measurement will reflect the period following baseline (when individuals participating in TEAM Uganda will have just completed the "intensive" group sessions), and it will be used to assess for within-subject differences from baseline to 13 weeks and 13 weeks to 6-month periods. This will allow for a greater understanding of the time course for when the expected reductions occur in the TEAM Uganda group, which we expect to mostly occur after the "intensive’ group sessions have ended. We will analyze the change from baseline to 13 weeks and analyze contrasts within the mixed model framework.

These analyses will compare two groups (TEAM intervention vs. enhanced treatment as usual (ETAU)) across three-time waves of systolic B.P., serum cholesterol, and serum HbA1c. When using a repeated measures analysis of variance (RMANOVA), we not only assess mean differences across time but also group differences, as well as the interaction of time X group, which will allow us to test the trend of the means over time between the two groups. The RMNOVA, not only can be used to determine if there are mean differences across the three time periods, it can also utilize orthogonal polynomial contrasts to determine linear and quadratic trends of the means across time. A linear trend is indicated if there is a steady increase or decline in scores from the first-time wave to the third time wave. A quadratic trend is indicated if there is a change in direction based on scores across the time waves.

We will also conduct exploratory analyses testing for the moderation of the explanatory variables in the primary mixed model. Mediation analyses also will be explored. Potential mediators will include the dose of TEAM session exposure, medication prescription/access, and adherence to a heart-healthy diet. Variables with (change) values that appear to be associated with a change in systolic B.P. levels and that appear to differ by treatment will be considered further as mediator variables, single mediator analyses, as well as multiple mediator models will be explored. These analyses will involve the treatment variable as the predictor of interest, potential mediators, and change in systolic B.P. values as the outcome of interest. If mediation is identified, bootstrapping will be used to identify the estimated indirect effects’ standard errors.

#### Missing data

Strategies to minimize loss to follow-up are outlined in Form E, specifically the description of our Recruitment and Retention Plan. Data that remain missing despite our retention efforts will be accommodated in our analyses and their impact evaluated through sensitivity analyses. The models we propose can be estimated without bias under the missing at random (MAR) assumption and provide valid analysis as long as covariates associated with missingness (If any) are included in the mixed model. To assess which covariates may be associated with missing outcome data, we will create binary indicators of whether the outcome was missing (= 1) or not (= 0). If a covariate is correlated with missingness at r >0.40 and is correlated at r >0.40 with the original response variable, it will be included in the analysis as an auxiliary correlate. We will conduct assessment of the missing at random (MAR) assumption by pattern mixture models that relax the missing at random assumption, while analyzing the sensitivity of treatment by time interaction effects. We will also consider using Full Information Maximum Likelihood models in presence of incomplete data.

#### Sample size estimation

Based on our preliminary mixed model preliminary data, we observed a difference in systolic blood pressure (BP) from baseline to 24 weeks of 13.22 (SD±25.84) mmHg and within a subject correlation parameter value of 0.57. We conservatively estimate our sample size, based upon a difference of 10 mmHg, the magnitude of change in B.P. across a large sample with varying baseline blood pressure levels and comorbidities [31]. Our projected sample size is n = 246, with 123 participants per arm, assuming a 25% attrition and a type I error level of 0.05 and a power of 0.80.

### Qualitative data analysis plan

#### Stage 1

Two qualitative researchers will be used to ensure standardization of qualitative analysis. The qualitative team will first independently review each transcript and highlight significant statements, sentences, or quotes. Based on the review of the independently derived statements, the team will develop consensus-based *"clusters of meaning" [32]* or relevant *"themes and categories"* [33]. Each researcher will further code each document independently and iteratively until no new insights emerge. Initial codes will be recorded using *NVivo*. These entries will be elaborated on as coding progress. The qualitative researchers will then construct a consensus-based coding dictionary that includes mutually exclusive definitions for each code. This coding structure will be reviewed after a preliminary analysis of a sub-sample of transcripts, and the dictionary will be refined through comparison, categorization, and discussion [33, 34]. The refined codes will then be applied to the transcripts, with coding decisions recorded electronically using NVivo. Both qualitative researchers will code all transcripts. In our previous research, checks for inter-rater consistency using Cohen’s Kappa [35] yielded Kappa ≥ .90, which was considered excellent agreement.

#### Stage 2

*NVivo* will retrieve all segments of text attached to a particular code to create code-based files across all respondents. The qualitative team will further elaborate, refine, and differentiate the codes and identify similarities and differences through the comparison of respondents. Emergent observations will be recorded in theoretical memos using *NVivo’s* Project Document feature. This engagement process with the data and iterative discussions will be repeated until all discrepancies are resolved, and no new insights emerge.

### Data and Safety Monitoring Board (DSMB) and safety review plan

Experts, who are not members of the study team, will review and evaluate the accumulated data for participant safety, adverse events, study conduct and progress, at minimum, every 12 months. Ad-hoc meetings might be called to evaluate unanticipated serious adverse events or any other urgent issues that are relevant and which might occur during the course of the study. The DSMB will be comprised of two clinicians with stroke expertise at the Uganda site; a faculty member/clinician with stroke expertise at the US site, and a biostatistician at the US site who are all not part of the study team, but have extensive experience with federally funded research. The DSMB communication and oversight will be accomplished via telephone, SKYPE, or email communication for issues that need more immediate attention.

As noted above, progress reports, including patient recruitment, retention/attrition, and adverse events will be provided to the DSMB at least annually for independent review. There are no specific study stopping rules as the intervention arm will receive self-management information that will help teach and support individuals who are at high risk for stroke in better managing their health. There will be an interim analysis conducted once half of all trial participants have completed the 6 -month outcome evaluation point. The DSMB will review the annual plan on all 5 points outlined above, as well as the interim analysis, and make recommendations to the appropriate regulatory agencies (IRB, NIH) concerning continuation, modification or termination of the study. The PI will provide a copy of all DSMB reports to the appropriate IRB and NIH on an annual basis.

### Ethics and dissemination

The study will be conducted according to the Helsinki Declaration [36], the NIH Human Subjects guidelines, and the International Conference on Harmonization E6 Guideline for Good Clinical Practice [37]. Approvals from local leaders will be sought before the study activities commence in the proposed areas. The study nurses will obtain informed consent from potential participants before study procedures are initiated. A copy of the consent form will be given to the study participant. Patient identifiers at the analytic level will not be the same as the patient’s clinic medical record number. The files that link the patient identifiers to the study numbers will be kept in locked cabinets in the study staff offices. Only aggregate data will be presented or published, and will be presented such that individual patients cannot be identified. This protocol version 2 dated, 25^th^ August 2020 was approved by the School of Medicine, Research and Ethics Committee (SOMREC) on 1^st^ October 2020; Rec Ref: 2020–179 and UNCST; HS1094ES, Case Western Reserve University IRB; STUDY20200882. The results of this study will be submitted for publication in peer-reviewed journals and the key findings will be presented at national conferences.

## Discussion

Despite the tremendous need, there are few practical and widely implemented care approaches that target the stroke burden in Uganda and other SSA countries [38]. While some individuals at high risk for stroke know their risk factors, they may not know how to address risk given their circumstances [39]. Sarfo and colleagues [38] are testing whether an m-Health technology-enabled, nurse-led, multilevel integrated approach effectively improves blood pressure among Ghanaian stroke patients. Kamwesiga and colleagues conducted a qualitative evaluation to characterize mobile phone communication’s potential utility to facilitate stroke patient recovery in Uganda [40].

While there are strengths to the approaches that exist in the limited literature, including use of mobile technology and a focus on the individuals at risk for recurrence, these approaches do not tap into the power of patients to become engaged as peers and educators, use family support networks, or substantively address other lifestyle factors for stroke risk. The Stroke Minimization through Additive Anti-atherosclerotic Agents in Routine Treatment (SMAART) trial conducted in SSA seeks to assess whether a polypill containing three antihypertensive agents can impact a stroke vascular biomarker (carotid intimal thickness) [41, 42]. However, medications are likely to work best if individuals understand the role of medications and take them in the context of a whole-person approach to risk reduction. An approach that engages people at risk for stroke and includes key social and cultural elements may be particularly salient for future generalizability and scale-up. The results of this randomized controlled trial should provide further evidence regarding TEAM self-management interventions and their ability to reduce the stroke risk.

## Abbreviations

BP: Blood pressure
ETAU: Enhanced Treatment As Usual
LMICs: low-middle income countries
NCDs: non-communicable diseases
PEDs: peer educator dyads
RCT: randomized controlled trial
SAB: stakeholder advisory board
TEAM: TargetEd MAnageMent Intervention program
